# Quantitative measurements of adaptive bone remodeling around the cemented Zimmer® segmental stem after tumor resection arthroplasty using dual-energy x-ray absorptiometry

**DOI:** 10.1186/s12891-021-04395-2

**Published:** 2021-06-05

**Authors:** Christina Enciso Holm, Peter Horstmann, Michala Skovlund Sørensen, Karen Dyreborg, Michael Mørk Petersen

**Affiliations:** Department of Orthopedic Surgery, Rigshospitalet, University of Copenhagen, Blegdamsvej 9, DK-2100 Copenhagen Ø, Denmark

**Keywords:** Tumor prostheses, Stress shielding, Bone mass density, Dual-energy X-ray absorptiometry

## Abstract

**Background:**

Limb salvage surgery (LSS) is the preferred method for treatment of patients with sarcomas and to a greater extent also to patients with metastatic bone disease. The aim of the present study was to evaluate the adaptive remodeling of the periprosthetic cortical bone after insertion of a tumor prosthesis with cemented stem.

**Methods:**

A prospective study of 21 patients (F/M = 12/9), mean age 55 years (range 15–81) with metastatic bone disease (*n* = 9), sarcomas (*n* = 8) or aggressive benign tumors (*n* = 4) who underwent bone resection due to a tumor, and reconstruction with a tumor-prosthesis (Zimmer® Segmental 130 mm straight fluted cemented stem with trabecular metal (TM) collars) in the proximal femur (*n* = 10), distal femur (*n* = 9) or proximal tibia (*n* = 2). Measurements of bone mineral density (BMD) (g/cm^2^) were done postoperatively and after 3, 6, and 12 months using dual-energy X-ray absorptiometry. BMD was measured in 4 regions of interest around the cemented stem and in one region of interest 1 cm proximal from the ankle joint of the affected limb and measurement of the contralateral ankle was used as reference. Repeated measures ANOVA and students paired t-test was used to evaluate BMD changes over time.

**Results:**

At 1-year follow-up, BMD decreased compared to baseline in all four regions of interest with a statistically significant bone loss of 8–15%. The bone loss was most pronounced (14–15%) in the 2 regions of interest closest to the trabecular metal (TM) collar and lowest (8%) adjacent to the tip of the stem.

**Conclusion:**

After 1 year the decrease in bone mineral density of the ankle on the affected limb was 9% and the contralateral ankle was close to baseline, thus suggesting that the periprosthetic bone mineral density changes during follow-up, mainly are caused by stress shielding and immobilization.

**Trial registration:**

The study was approved by the Scientific Ethical Committee of the Capital Region of Denmark (J. No. H-2-2014-105) and the Danish Data Protection Agency (J. No.:2012–58-00004).

## Background

Limb sparing surgery (LSS) is today the preferred surgical treatment of bone sarcomas in the lower extremities [1]. The same patient survival is reported if LSS is performed in the majority of cases instead of amputation [2]. Following bone tumor resection, reconstruction of the affected limb is usually done using tumor prostheses in order to save the function of the affected limb. The same technique (LSS and tumor prostheses) are increasingly applied in the treatment of patients suffering from bone destruction because of metastatic bone disease (MBD) [3].

Modern tumor prostheses are attached to the bone using cemented or uncemented intramedullary stems. After implantation of the stem, periprosthetic loss of bone stock in close relation to the stem is to be expected for various reasons such as the bone reaction to the operative trauma, postoperative immobilization, chemotherapy, and stress shielding [4–6]. Compared to ordinary primary hip or knee arthroplasty, LSS and reconstruction using tumor prostheses causes greater operative trauma and prolonged rehabilitation. Furthermore, patients will often be in need of chemotherapy prior and after surgery. Stress shielding after primary hip arthroplasty is well known and occurs around the well-fixed un-cemented and cemented stems and is characterized by thinning of the compact diaphyseal bone adjacent to the stem [7]. Stress shielding represents a considerable clinical problem after insertion of tumor prostheses due to increased risk of periprosthetic fracture, and also greater complexity in case of revision [8].

Stress shielding after joint replacement surgery has previously been reported using quantitative densitometric techniques in several studies [9–11]. Dual-energy x-ray absorptiometry (DXA) [12, 13] has been used extensively for quantitative and precise measurements of changes in bone mineral density (BMD) in close relation to both cemented and un-cemented orthopedic implants [14–16]. Only three studies [5, 8, 17] of partly cross-sectional design (with no immediate postoperative measurements performed) or with a very limited number of patients with inserted tumor prostheses because of malignant bone tumor resection have been published and no real prospective quantitative measurements of the adaptive bone remodeling around the fixation stems of tumor prostheses exist.

In that perspective the aim of the present study was, in a prospective design using DXA, to quantitatively measure the adaptive bone remodeling around the intramedullary 130 mm Zimmer® Segmental straight fluted cemented stem in patients with malignant bone tumors receiving tumor prostheses. We hypothesize that the use of trabecular metal (TM) collars together with the intramedullary 130 mm cemented Segmental stem will secure an optimal stem fixation, thus reducing stress shielding of the periprosthetic cortical bone compared to the sparse previous reports.

## Material and methods

### Patient population

**Fig. 1:**
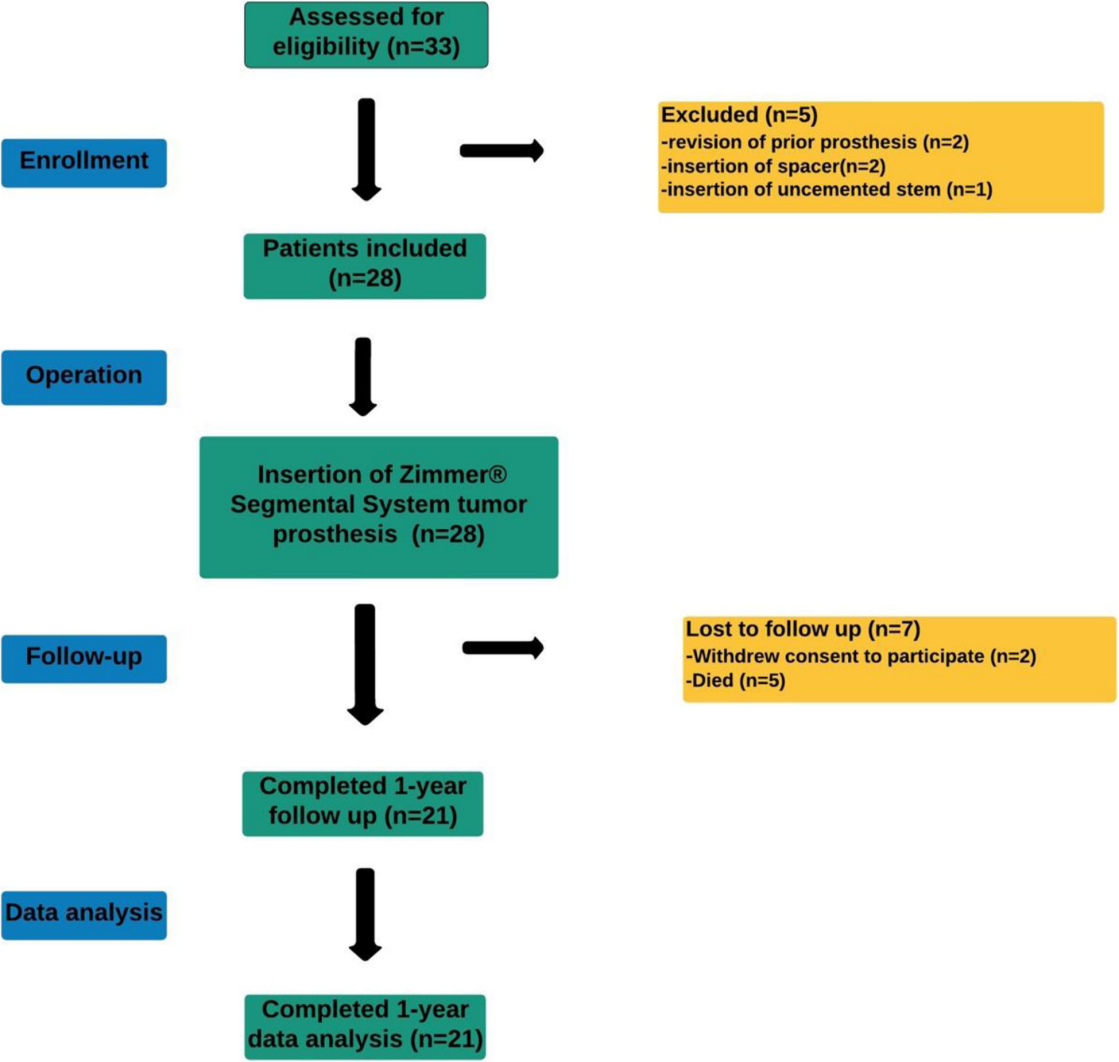
Flow chart. Enrollment, follow-up, and data analysis

**Fig. 2:**
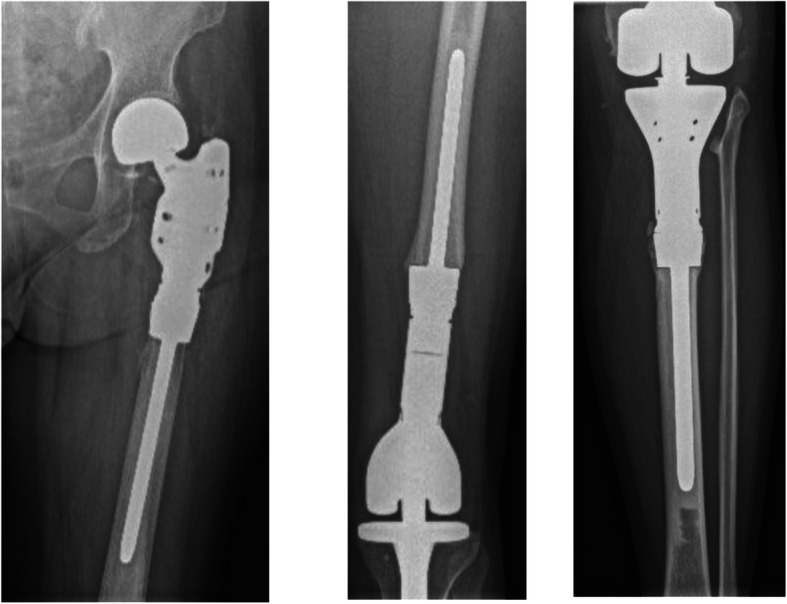
Radiographs of proximal femur tumor arthroplasty, Cemented Zimmer® Segmental stem (left). Radiographs of distal femur arthroplasty, Cemented Zimmer® Segmental stem (middle). Radiographs of proximal tibia arthroplasty, Cemented Zimmer® Segmental stem (right)

Between January 1, 2015 and July 1, 2018, 33 patients who underwent bone tumor resection with LSS and reconstruction with a Zimmer® Segmental System tumor prosthesis (Zimmer Biomet) in the lower extremities, were evaluated for inclusion in the study (Fig. 1). It was predefined to exclude patients with age < 15 years, patients with diseases severely affecting the bone metabolism and patients with expected survival below 1 year (estimated by the surgeon and the investigators). Furthermore, patients with infection, including osteomyelitis and sepsis were excluded. Also, patients with alcohol or drug abuse were excluded. Patients with pregnancy were excluded. Lastly, we excluded patients with lack of compliance or patients considered unable to understand the information in patient-papers or who did not want to participate in the study. Twelve patients were excluded for various reasons, and 21 patients (F/M = 12/9, mean age 55 years) diagnosed with a primary bone tumor (*n* = 6), an aggressive benign tumor (*n* = 4), myelomatosis (*n* = 2) or MBD (*n* = 9) (Table 1) completed 1-year follow-up (Fig. 1**).** All surgeries were carried out by, or under supervision of, an experienced tumor joint replacement surgeon at a tertiary referral center for orthopedic oncology. All reconstructions were done using the Zimmer® Segmental tumor prostheses with an intramedullary 130 mm straight fluted stem for cementation and a TM collar (Fig. 2). Patients were mobilized with full weight-bearing using crutches the day after surgery. Clinical evaluation of the treatment was conducted by using the Enneking score (MSTS-score) [18] after 3, 6 and 12 months.

**Table 1:** Baseline data of the patients (*n* = 21) that completed 1-year follow-up

Variable	Level	Total (%)
**Gender**	*Female*	*12 (57%)*
	*Male*	*9 (43%)*
**Age (years)**	*Mean (range)*	*55 (15–81)*
**Resection (cm)**	*Mean (range)*	*15 (10–24)*
**Resection site**	*Proximal femur*	*10 (48%)*
	*Distal femur*	*9 (43%)*
	*Proximal tibia*	*2 (10%)*
**Pathology**	*Metastasis*	*9 (43%)*
	*Giant Cell*	*4 (19%)*
	*Chondrosarcoma*	*2 (10%)*
	*Myelomatosis*	*2 (10%)*
	*Osteosarcoma*	*2 (10%)*
	*Myxoid liposarcoma*	*1 (5%)*
	*Desmoplastic fibroma*	*1 (5%)*

### DXA evaluation

BMD (g/cm^2^) of the periprosthetic bone of the femur or tibia around the stem and adjacent to the TM collar was measured by DXA using a Norland XR-46 scanner (scan resolution 0.5 × 0.5 mm, scan speed 45 mm/s) postoperatively and after 3, 6, and 12 months. All patients were placed supine with the femur in neutral rotation during scanning. On the computerized scan-plots, we selected three regions of interest (ROI) around the stem in the femoral or tibial bone and one ROI adjacent to the TM collar for measurements of local changes in BMD over time around the fixation stem: a 2.5–3 cm long area for the bone adjacent to the TM collar (ROI 1), a 5 cm area comprising the middle part of the stem (ROI 2), a 5 cm area comprising the distal part of the stem (ROI 3) and a 3 cm long area comprising the bone adjacent to the tip of the stem (ROI4) (Fig. 3). A custom-made metal exclusion software facility, which allows a variable threshold for metal exclusion, was used for scan analysis. The threshold (range: 4.0 g/cm^2^–6.0 g/cm^2^) used, was not the same in all patients but in each individual, it was kept the same. The precision of the BMD measurements was calculated from double measurements of 6 patients, and we found a mean coefficient of variation (CV) of 5% (range 0.8–16%), 3% (range 0.1–12%), 2% (range 0.4–8.5%), and 3% (range 0.7–6%) for ROI1, ROI2, ROI3, and ROI4 respectively.

**Figure:**
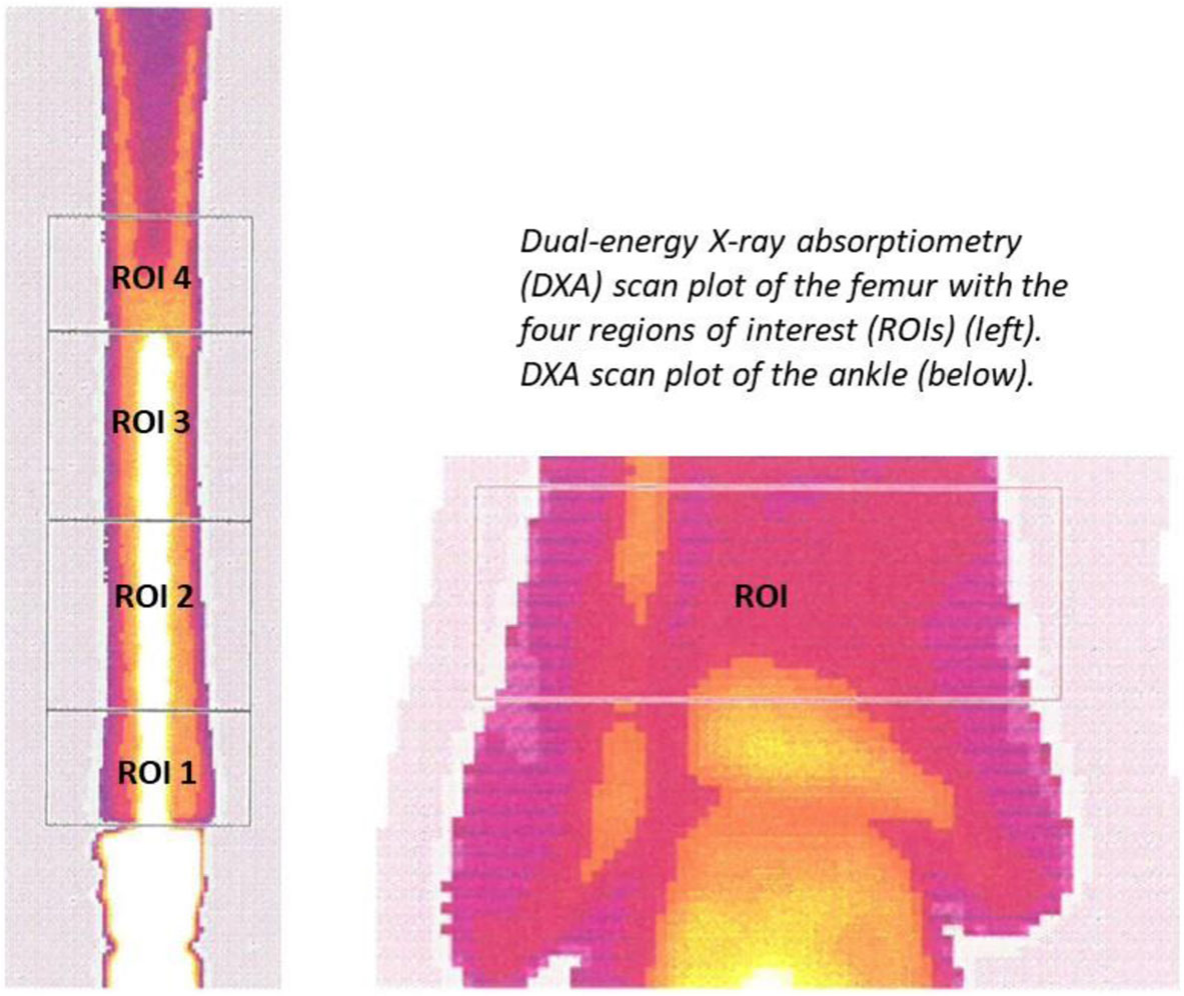
Fig. 3

Using the same DXA technique (scan resolution: 1.0 × 1.0 mm; scan speed: 45 mm/s), we also performed scans of the ankle of the operated side and the contralateral non-operated side postoperatively and after 3, 6, and 12 months to address a potential decrease in BMD caused by immobility or general decrease. BMD was measured in a 2-cm long ROI located 1 cm proximal from the ankle joint (Fig. 3). These scans were performed as previously described and the precision error for measurements of BMD in this ROI is very low [19].

### Statistics

The BMD data was considered normally distributed. All changes in BMD over time were analyzed using repeated measures ANOVA and students paired t-test for comparison of the step-wise BMD changes over time compared to the first postoperative scanning. *P*-values below 0.05 were considered significant. Precision of the BMD measurements was evaluated by calculation the coefficient of variation (CV = (standard deviation (SD) / mean) × 100%). All data is presented as mean (SD or range). The statistical analysis was performed using software R (R foundation, Vienna, Austria).

## Results

### Clinical results

The mean MSTS score was 17 (5-29) after 3 months. The score did not change during the follow-up, and it was 18 (4-30) after 12 months representing a mean score of 59%. After 3 and 6 months, the highest score was in the emotional acceptance category (mean score: 3.8) and lowest in the function category (mean score 1.9). One year after surgery, patients scored highest in the walking category (3.6) and lowest in function (2.0).

### BMD changes around the stem

We found a significant decrease in periprosthetic BMD during 1-year follow-up in all ROI’s, however, in ROI2 and ROI3 statistical significance was only obtained using t-test (0–12 months) and not by the ANOVA analysis (Table 2). The greatest reduction in BMD, 1 year after surgery, was in ROI2 (15%). Within the first 3 months, ROI2 showed the highest decrease in BMD of 8% (*p* = 0.366) compared to baseline. From 3 to 6 months, BMD increased close to baseline in ROI2 (− 0.4%) followed by a further decrease in BMD after 12 months of 15% below baseline (*p* = 0.003). In ROI1, adjacent to the TM collar, the BMD progressively decreased from 6% within the first 3 months until 14% below baseline after 1 year (*p* = 0.004). In ROI3, closest to the tip of the stem, BMD decreased 6% after 3 months and gradually decreased further to 11% below baseline after 1-year of follow-up (*p* = 0.005). ROI4 adjacent to the tip of the stem showed the lowest decrease in BMD within all follow-up measures although statistically significant after 12 months (8%, *p* < 0.0001).

**Table 2:** Mean (SD) BMD (g/cm2) in the 4 ROIs around the stem and in both ankles (operated and non-operated contralateral legs)

Follow-up	Postoperative (***n*** = 21)	3 months (***n*** = 18)	6 months (***n*** = 21)	12 months (***n*** = 21)	***p***-value^**#**^ 0–12 months (***n*** = 18)
**ROI1**, BMD	2.186 (0.38)	2.056 (0.48)	1.990 (0.46)	1.874 (0.27)	0.037
ΔBMD%		−6%	−9%	− 14%	
*p* values (stepwise)* CI (95%)		0.285 (−0.10–0.33)	0.092 (− 0.04–0.43)	0.004 (0.11–0.52)	
**ROI2**, BMD	2.248 (0.41)	2.075 (0.53)	2.238 (0.57)	1.914 (0.30)	0.071
ΔBMD%		−8%	−0.4%	−15%	
*p* values (stepwise)* CI (95%)		0.366 (−0.15–0.37)	0.95 (− 0.28–0.30)	0.003 (0.13–0.54)	
**ROI3**, BMD	2.215 (0.43)	2.075 (0.49)	2.071 (0.38)	1.978 (0.3)	0.223
ΔBMD%		−6%	- 7%	−11%	
*p* values (stepwise)* CI (95%)		0.438 (−0.16–0.35)	0.117 (− 0.04–0.33)	0.005 (0.08–0.39)	
**ROI4**, BMD	2.080 (0.42)	2.047 (0.44)	1.948 (0.45)	1.923 (0.45)	0.009
ΔBMD%		−2%	−4%	−8%	
*p* values (stepwise)* CI (95%)		0.356 (−0.04–0.11)	0.079 (−0.01–0.18)	< 0.0001 (0.09–0.22)	
**Ankle operated**, BMD	0.751 (0.15)	0.7048 (0.14)	0.7049 (0.17)	0.681 (0.16)	< 0.001
ΔBMD%		−6%	−6%	−9%	
*p* values (stepwise)* CI (95%)		0.008 (0.01–0.06)	0.008 (0.02–0.09)	< 0.001 (0.05–0.11)	
**Ankle contralateral**, BMD	0.806 (0.19)	0.793 (0.18)	0.814 (0.25)	0.788 (0.17)	0.322
ΔBMD%		−2%	+ 1%	−2%	
*p* values (stepwise)* CI (95%)		0.12 (−0.01–0.05)	0.90 (−0.08–0.07)	0.12 (− 0.01–0.05)	

^*^students paired t-test, ^#^repeated measures ANOVA

**Fig. 4:**
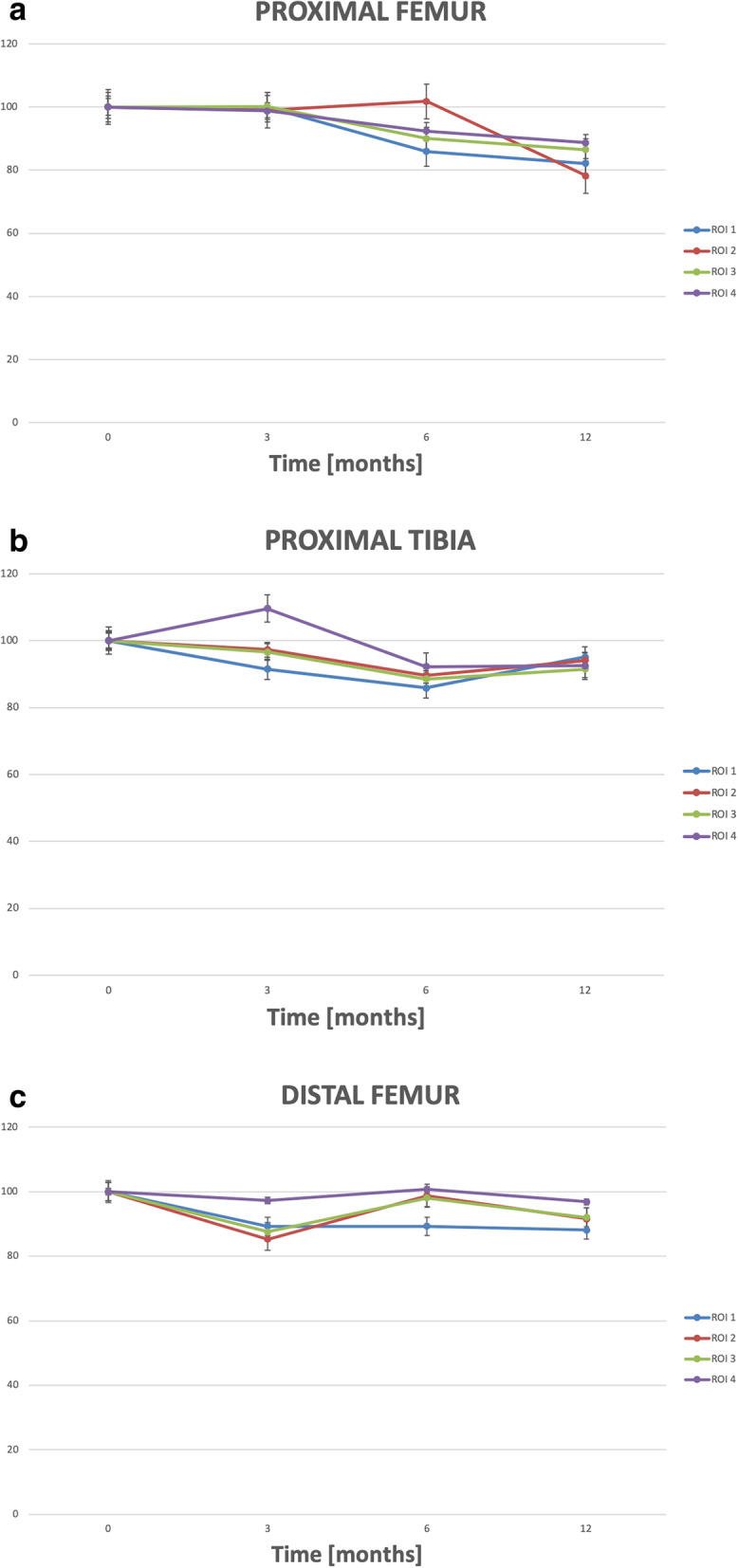
**a**. Mean (SE) BMD changes in percent of the 4 ROI in proximal femur. **b**. Mean (SE) BMD changes in percent of the 4 ROI in proximal tibia. **c**. Mean (SE) BMD changes in percent of the 4 ROI in distal femur

We found the largest reduction in BMD after 12 months around proximal femoral stems (11–18%) (Table 3) (Fig. 4a) when comparing with distal femoral stems (3–12%) (Table 4) (Fig. 4b) and proximal tibia (5–9%) (Table 5) (Fig. 4c). Due to the low number of patients and risk of type 2 errors, no statistical comparison was performed between anatomical sites.

**Table 3:** Mean (SD) BMD (g/cm2) in the 4 ROIs around the stem in proximal femur

	BMD (g/cm^**2**^) Postoperative (***n*** = 10)	BMD (g/cm^**2**^) 0–3 months (***n*** = 9)	BMD (g/cm^**2**^) 0–6 months (***n*** = 10)	BMD (g/cm^**2**^) 0–12 months (***n*** = 10)
**ROI1**	2.272	2.269	1.949	1.866
Range	1.04–3.57	1.607–3.628	1.110–2.586	1.431–2.591
SD	0.661	0.648	0.445	0.386
ΔBMD%		0	−14	−18
**ROI2**	2.333	2.310	2.374	1.824
Range	1.782–3.408	1.697–3.796	1.782–3.847	1.152–2.317
SD	0.452	0.658	0.624	0.352
ΔBMD%		−1	+ 2	−22
**ROI3**	2.247	2.251	2.023	1.943
Range	1.692–3.010	1.669–3.370	1.692–2.471	1.580–2.370
SD	0.0.397	0.561	0.285	0.307
ΔBMD%		0	−10	−14
**ROI4**	2.172	2.145	2.007	1.926
Range	1.348–3.231	1.288–3.045	1.143–2.962	1.068–2.877
SD	0.507	0.582	0.524	0.513
ΔBMD%		−1	−8	−11

**Table 4:** Mean (SD) BMD (g/cm2) in the 4 ROIs around the stem in distal femur

	BMD (g/cm^**2**^) Postoperative (***n*** = 9)	BMD (g/cm^**2**^) 0–3 months (***n*** = 8)	BMD (g/cm^**2**^) 0–6 months (***n*** = 9)	BMD (g/cm^**2**^) 0–12 months (***n*** = 9)
**ROI1**	2.163	1.931	2.123	1.905
Range	1.772–3.191	1.675–2.195	1.574–3.409	1.687–2.143
SD	0.42	0.16	0.52	0.16
ΔBMD%		−11	−11	−12
**ROI2**	2.257	1.926	2.231	2.067
Range	1.955–3.522	1.087–2.170	1.898–3.133	1.825–2.251
SD	0.48	0.35	0.37	0.14
ΔBMD%		−15	−1	−8
**ROI3**	2.297	2.012	2.254	2.115
Range	1.955–3.514	1.120–2.625	1.955–2.961	1.893–2.414
SD	0.48	0.42	0.31	0.16
ΔBMD%		−12	−2	−8
**ROI4**	2.100	2.043	2.115	2.034
Range	1.675–2.486	1.631–2492	1.840–2.365	1.602–2.443
SD	0.23	0.27	0.18	0.27
ΔBMD%		−3	+ 1	−3

**Table 5:** Mean (SD) BMD (g/cm2) in the 4 ROIs around the stem in proximal tibia

	BMD (g/cm^**2**^) Postoperative (***n*** = 2)	BMD (g/cm^**2**^) 0–3 months (***n*** = 2)	BMD (g/cm^**2**^) 0–6 months (***n*** = 2)	BMD (g/cm^**2**^) 0–12 months (***n*** = 2)
**ROI1**	1.858	1.699	1.595	1.767
Range	1.825–1890	1.657–1.741	1.486–1.703	1.752–1.782
SD	0.05	0.06	0.15	0.02
ΔBMD%		−9	−14	−5
**ROI2**	1.775	1.727	1.591	1.671
Range	1.743–1.807	1.708–1.745	1.507–1.674	1.665–1.676
SD	0.05	0.03	0.12	0.01
ΔBMD%		−3	−10	−6
**ROI3**	1.683	1.626	1.489	1.540
Range	1.651–1.714	1.594–1.657	1.417–1.561	1.449–1.631
SD	0.04	0.04	0.10	0.13
ΔBMD%		−3	−12	−9
**ROI4**	1.527	1.674	1.408	1.412
Range	1.449–1.605	1.475–1.872	1.389–1.427	1.319–1.505
SD	0.11	0.28	0.03	0.13
ΔBMD%		+ 10	−8	−8

### BMD changes of the ankles

After 3 months, the BMD decreased by 6% (*p* = 0.008) in the operated ankle followed by a temporary plateau after 6 months, and finally at 1-year of follow up, the BMD loss in the operated ankle reached 9% below baseline (*p* = < 0.001). We found an initial minor decrease of 2% (*p* = 0.12) in BMD in the non-operated ankle after 3 months and it stayed approximately at that level throughout the study period (Table 2).

## Discussion

During the first year after surgery, significant BMD changes were seen in all four ROI around the 130 mm cemented stem of the Zimmer® Segmental tumor prosthesis ending with a significant bone loss after 1 year of 8–15%. The bone loss was most pronounced (14–15%) in the 2 ROIs closest to the TM collar and lowest (8%) adjacent to the tip of the stem.

To our knowledge, there exist no previous reported longitudinal results of the periprosthetic bone remodeling after resection and reconstruction with the cemented Zimmer® Segmental tumor prosthesis. Only a few studies have investigated the periprosthetic bone remodeling after insertion of a tumor prosthesis [5, 8, 17]. As in the present study, Lan et al. [8] and Andersen et al. [17] found a further reduction in bone mineral with increased distance from the distal part of the stem towards the extension pieces, or prostheses, corresponding to the Gruen Zones 1, 2, 6 and 7 [20, 21]. The same pattern in BMD changes along the stem, as demonstrated by Lan et al. [8], was found in a cross-sectional study with a mean time of 31.8 months after surgery, using the contralateral leg as reference. However, the evaluation of BMD changes over time by Lan et al. [8] was based upon measurements in one selected ROI which limits comparison. Vennesma et al. [20] demonstrated that to obtain exact measurements of BMD changes after surgery, the operated side should always be reference and patients should be followed prospectively. Likewise, Kröger et al. [15] demonstrated that there are local differences in BMD between limbs and stated that BMD measurements years after surgery compared with contralateral values are invalid. The absolute and relative changes in BMD across all ROI within the present follow up are comparable to the remodeling around stems used in other tumor prostheses as demonstrated by Andersen et al. [17]. Davis et al. [5] evaluated bone remodeling around the Kotz Modular Femur Tibia Reconstruction with a mean of 90.2 months after surgery and their results indicated that BMD reached a plateau. However, their study was cross-sectional using the contralateral limbs as reference and an interstudy comparison is therefore questionable.

The pattern in bone remodeling along the Zimmer® Segmental stem is corresponding to other findings after both cemented and uncemented primary hip arthroplasty [9, 21–23]. Bone remodeling and bone resorption adjacent to the proximal part of the stem is caused by distal transfer load of the prostheses due to the greater stiffness of the stem. Thus, the periprosthetic bone close to the artificial joint itself is more prone to stress shielding.

Several studies investigating primary hip arthroplasty reported a pronounced periprosthetic loss in BMD around the cemented and uncemented femur stem within the first 3 months after surgery followed by an increase or plateau after 6 month [15, 20]. The adaptive changes in bone remodeling caused by the surgical trauma to the bone after arthroplasty has been suggested to be long lasting despite increased postoperative activity [12, 24]. However, Brodner et al. [25] and Huang et al. [26] found increased BMD in the distal Gruen zones after 5 and 3 year follow up respectively and Korovessis et al. [27] found increased BMD at the greater and minor trochanter after 4 years follow-up. Our results indicate a progressive remodeling and loss in BMD after 1 year.

When comparing anatomic sites we found the most pronounced loss in BMD around the stems in proximal femur. Due to the low number of patients with proximal tibia tumor prostheses, we found those results not sufficient for comparison. Only few studies have illuminated changes in BMD of the distal femur following a stemmed femoral implant. Jensen et al. [28] found a significant increase in periprosthetic BMD during the first 6 months after surgery with the largest increase adjacent to the most proximal part of the stem. Our findings did not demonstrate the same pattern in BMD changes along the stem. However, the implants examined in the study by Jensen et al. [28] were not tumor-prostheses but regular stemmed revision total knee arthroplasty femoral components inserted without bone resection. In a finite element study, Van Lenthe et al. [29] studied bone loss and remodeling patterns of four femoral components: two primary TKAs and two stemmed revision prostheses with stem diameter of, respectively, 18 and 12 mm. Van Lenthe et al. [29] found the same pattern of bone resorption along the stem as in present study i.e. increased periprosthetic bone loss in the proximal, part of the stem, decreasing towards the distal part of the stem. We suggest the findings by Jensen et al. [28] are due to the described pre-operative immobilization of their patients followed by increased postoperative mobility. Patients in present study suffered to a large extend from pre-operative almost normal mobilization followed by prolonged post-operative immobility and sometimes chemotherapy. Nevertheless the indicated reduced loss in BMD around the distal femur stems compared to proximal femur stems may indicate the different mechanical load between sites.

Even though we used cemented fixation for all our prostheses with immediate weight bearing, the demonstrated progressive bone remodeling after 1-year could partly be explained by the well known required prolonged rehabilitation and immobilization after inplantation of tumor prostheses. This is due to prolonged surgery time and extensive loss of tissue. Furthermore, loss of bone stock in relation to chemotherapy is well described [4] and given the mean age in the present cohort, the well known age-related decay [30] in BMD will further affect the risk of progressive bone resorption after surgery.

It is well known from primary hip or knee arthroplasty that lesser stem stiffness, shorter stems and also coating may contribute to retain normal load transfer, and thus enhance bone preservation [14, 22, 23]. The various long-term follow-up results in periprosthetic BMD shows that adaptive bone remodeling after surgery also may contribute to better fixation as opposed to loosening and that it could depend on fixation method of the prostheses due to advantageous distribution and transmission of load. We speculate that the relative slow decrease in BMD until 1-year after surgery in all our ROI partly could be explained by the intended fixation of the TM collar with less load transfer to the tip of the stem and hence reduced stress shielding adjacent to the joint. However, inter study comparison in general is difficult due to differences in measurement of BMD, prostheses, methods of fixation and also patient cohort with regards to age, gender and comorbidity.

The average MSTS score was 22.3 (range: 14–30) 1 year after surgery. The patients scored highest in the walking and gait (average: 4.3) categories and lowest in function and supports (average: 3.3) categories.

The average MSTS score is slightly poorer compared to other studies evaluating tumor prostheses [10, 31]. Due to the need for prolonged rehabilitation after insertion of tumor prostheses, we suggest that the difference is partly caused by the relatively short follow up in our study compared to other studies. Also, we speculate that the MSTS score reflects that our cohort also comprised patients with MBD, which is often a group of patients in poor general health condition. Nevertheless, we find our results comparable to the 1-year evaluation by Andersen et al. [17].

To assess to what extend the periprosthetic changes in BMD were caused by stress shielding, immobilization or a general decrease in BMD for other causes, we performed DXA scans of both ankles. The immobilization of the operated limb is considered to be reflected by the decrease in BMD of the affected ankles. After 1-year, the decrease in BMD of the operated ankle was 9% and the non-operated ankle was close to baseline (2%). These findings indicate that the periprosthetic BMD changes during follow-up are caused by stress shielding combined with immobilization and to a lesser extend a general decrease in BMD.

We found a precision of BMD measurement of CV 2–5% which is slightly higher compared to Andersen et al. [17] evaluating the uncemented proximally Hydroxyapatite-Coated femur stem. This could partly be explained by the bone-cement interface in our measurements. Lan et al. [32] evaluated the Kotz Modular Femoral Tibial Reconstruction stems with screw fixation and found CV comparable to ours despite the fact, that they evaluated uncemented stems. However, their measures are based upon smaller ROI and since Gehrchen et al. [33] demonstrated that lesser ROI is associated with poorer precision, the smaller ROI size therefore could be an explanation. Nevertheless, we find our CV comparable to previous findings of cemented hip and knee arthroplasty which has proven to be adequate values to detect small adaptive bone remodeling changes [15, 22, 34].

Some limitations need to be addressed. Our sample size is relatively small and non-randomized. However, to the best of our knowledge randomized controlled trials, to evaluate different implants and methods of fixation for these patients, is not an option. Also, repeated measures can be biased by outside factors including outcome during follow-up. In addition, in case of missing values, repeated measure ANOVA, excludes all data of the participant. Furthermore, repeated measures is well suited for small sample size and despite 7 patients lost to follow up, we have only few missing data of those who completed 1-year data analysis follow-up and all available data was used when performing post-hoc students paired t-test. Nevertheless, to the best of our knowledge present study demonstrates the largest sample size in a prospectively designed study evaluating bone remodeling around a tumor prosthesis with 1-year follow-up.

## Conclusion

In conclusion, we successfully evaluated the early adaptive bone remodeling around the cemented Zimmer® Segmental stem and the TM collar, used for reconstruction after tumor resection in the lower extremities. Our results indicated a slow progressive decrease in BMD of 8–15% after 1-year follow up, and the periprosthetic bone loss is considered (from the results of BMD measurements of the ankles) to be caused by a combination of stress shielding and immobilization.

## Data Availability

Due to the Danish law the datasets used and/or analyzed during the current study is not available from the corresponding author.

## Abbreviations

LSS: Limb sparing surgery
BMD: Bone mineral density
DEXA: Dual-energy x-ray absorptiometry
TM: trabecular metal
MBD: Metastatic bone disease
ROI: Region of interest
